# GeneTIER: prioritization of candidate disease genes using tissue-specific gene expression profiles

**DOI:** 10.1093/bioinformatics/btv196

**Published:** 2015-04-09

**Authors:** Agne Antanaviciute, Catherine Daly, Laura A. Crinnion, Alexander F. Markham, Christopher M. Watson, David T. Bonthron, Ian M. Carr

**Affiliations:** ^1^Section of Genetics, Institute of Biomedical and Clinical Sciences, School of Medicine, University of Leeds, St James’s University Hospital and; ^2^Yorkshire Regional Genetics Service, St James’s University Hospital, Leeds, UK

## Abstract

**Motivation:** In attempts to determine the genetic causes of human disease, researchers are often faced with a large number of candidate genes. Linkage studies can point to a genomic region containing hundreds of genes, while the high-throughput sequencing approach will often identify a great number of non-synonymous genetic variants. Since systematic experimental verification of each such candidate gene is not feasible, a method is needed to decide which genes are worth investigating further. Computational gene prioritization presents itself as a solution to this problem, systematically analyzing and sorting each gene from the most to least likely to be the disease-causing gene, in a fraction of the time it would take a researcher to perform such queries manually.

**Results:** Here, we present Gene TIssue Expression Ranker (GeneTIER), a new web-based application for candidate gene prioritization. GeneTIER replaces knowledge-based inference traditionally used in candidate disease gene prioritization applications with experimental data from tissue-specific gene expression datasets and thus largely overcomes the bias toward the better characterized genes/diseases that commonly afflict other methods. We show that our approach is capable of accurate candidate gene prioritization and illustrate its strengths and weaknesses using case study examples.

**Availability and Implementation:** Freely available on the web at http://dna.leeds.ac.uk/GeneTIER/.

**Contact:** umaan@leeds.ac.uk

**Supplementary information:** Supplementary data are available at *Bioinformatics* online.

## 1 Introduction

Current high-throughput sequencing methods used for disease gene discovery can generate very large volumes of data. While the extraction of non-synonymous, potentially deleterious variants can be easily automated, this often results in the identification of thousands of candidate disease genes. Since the experimental verification of an individual gene can be both difficult and time consuming, some method to prioritize the order in which such verification is sought is often employed. A common approach is to examine biological databases and literature for information pertaining to each candidate disease gene, in order to select the most promising genes. This can be both slow and error-prone, as the data are spread across multiple resources with no common structure. Nor can this type of analysis be quantified, since the selection is based solely on the subjective impressions of the researcher.

In light of these challenges, various computational gene prioritization methods have been proposed. These vary substantially in terms of both intended usage and underlying algorithms (reviewed in Tranchevent *et al.,* 2011). Generalized methods rely extensively on data mining of sequence information, gene expression datasets, functional annotations and/or protein–protein interactions. These data are most frequently employed in a ‘guilt-by-association’ framework, in which candidate disease genes are ranked based on the strength of relationships and/or similarities to genes already known to be linked with the disease. One of the major drawbacks of such approaches is the bias introduced toward better characterized genes and/or diseases. Thus, in particular where little prior knowledge about the disease and/or gene is available, accurate prioritization of putative disease genes remains a challenge.

The availability and wide coverage of experimental conditions of gene expression datasets might alleviate issues arising from incomplete data, as current technologies allow for the quantification of an entire transcriptome. Indeed, gene expression data are a crucial constituent of several web-based gene prioritization applications; however, it tends to be used in concert with other data types, such as pathway/interaction networks or functional annotations. Established web tools such as ToppGene (Chen *et al.,* 2009), Endeavour (Tranchevent *et al.*, 2008) and CANDID (Hutz *et al.*, 2008) employ a modular approach to prioritization, scoring candidates based on a consensus from multiple data sources. Even though it has been demonstrated that consensus methods are more accurate than approaches utilizing fewer data categories (Bornigen *et al.*, 2012), the former have been criticized for both the ‘guilt-by-association’ bias and failure to exploit the best performing methods for each component (Chen *et al.*, 2011).

More specialized algorithms often integrate gene expression data into a heterogeneous (Chen *et al.*, 2011, Ma *et al.*, 2007, Nitsch *et al.*, 2011) or homogenous (van Dam *et al.*, 2012) network, where distance between genes can be derived from and/or weighted by differential expression or co-expression values. Alternatively, some methods (Chen *et al.*, 2009, Masotti *et al.*, 2008, Seelow *et al.*, 2008) consider gene co-expression in a non-network context, utilizing common statistical vector correlation measures to rank candidate disease genes based on how well their expression patterns correlate with those of genes known to be directly or indirectly linked to the disease.

Fewer applications attempt to apply tissue-specific expression patterns for gene prioritization tasks. Endeavour (Tranchevent *et al.*, 2008) incorporates gene expression data from 79 normal human tissues found in Gene Expression Atlas, comparing gene expression between candidate and user-supplied seed genes across tissues. A recent update to PhenoDigm (Oellrich and Smedley, 2014 Smedley *et al.*, 2013) has incorporated tissue-specific, binary mouse gene expression data from 21 mouse tissues and derived phenotype-tissue associations in order to supplement its phenotype-based queries.

Nonetheless, none of these approaches distance themselves entirely from the ‘guilt-by-association’ approach. Here, we investigate the use of publicly available gene expression data as the sole means of prioritizing candidate disease genes. The resulting web application GeneTIER scores candidate disease genes based on the hypothesis that genes responsible for a tissue(s)-specific phenotype are expected to be more highly expressed in affected than unaffected tissues. GeneTIER depends on an extensive database that has been built using publicly available microarray and RNA sequencing datasets and is composed of several million expression values for numerous normal tissues. This enables the creation of a global, cross-tissue expression profile for each candidate disease gene, permitting expression profile-based prioritization without reliance on or requirement for other prior knowledge about the disease or candidate genes. GeneTIER should thus be suitable for prioritization of candidates for poorly characterized diseases.

## 2 Methods

### 2.1 Database

The Gene expression database contains 9 972 862 baseline gene expression values from microarray and RNA-Seq experiments, encompassing 140 different control, non-diseased mouse and human tissue types. The database was assembled from public domain sources, including datasets from Gene Expression Atlas (Petryszak *et al.*, 2014), RNA-Seq Expression Atlas (Krupp *et al.*, 2012), ArrayExpress (Rocca-Serra *et al.*, 2003) and Gene Expression Omnibus (Barrett *et al.*, 2009).

Microarray probe set to Ensembl gene transcript identifier mappings were downloaded from the Biomart resource (Kasprzyk, 2011). As per recommended practice, ambiguous data arising from microarray probes which hybridize to more than one distinct gene were discarded (Ramasamy *et al.*, 2008). Similarly, HGNC, Ensembl and Entrez and RefSeq gene identifiers were obtained from Biomart. UCSC gene names and exon boundary coordinates were downloaded using the UCSC Genome Browser’s ‘Table’ page (Karolchik *et al.*, 2004) using the hg19 human genome assembly. Mouse–human gene orthologs were downloaded from MGI (Bult *et al.*, 2008) and mapped using HomoloGene (Flicek *et al.*, 2014).

### 2.2 The gene prioritization algorithm

Candidate genes are ranked based on several factors derived from gene expression data. These comprise the levels of expression in the affected tissues; variance in expression across all tissues; and expression level differences between affected and unaffected tissues. The base score, $\left(S_{g}\right)$, for each candidate disease gene is calculated using Equation (1):

(1) $$S_{g}=\underset{t\epsilon T}{\sum }\{\begin{array}{cc} {\bar{z}}_{t} & \text{if}\;{\bar{z}}_{t}=0\\ {\bar{z}}_{t}\cdot \left(1+\text{ln}\frac{{\bar{z}}_{t}}{\overset{∼}{z}}\right) & \end{array}$$

where *t* is an affected tissue in a set of all affected tissues *T*; ${\overset{¯}{z}}_{t}$ is the mean of modified *z*-scores (see below) for tissue *t*; and $\overset{∼}{z}$ is the median modified *z*-score across all tissues. If gene expression in an affected tissue is greater than its baseline expression the natural logarithm ratio is positive; otherwise the value is negative. The value of $\left(S_{g}\right)$ is a fractional modifier, favoring genes which show elevated gene expression in disease-associated tissues, compared with tissues not linked to the disease phenotype, even if the expression value is relatively low. The score can be further adjusted for highly expressed genes which takes into account the level of variance in expression across all tissues in order to reduce the contention of highly ubiquitously expressed housekeeping genes. When included in the analysis, the results from human RNA sequencing, human microarray, mouse RNA sequencing and mouse microarray data are each considered separately, and combined to generate the final ranking score. In cases where incomplete data may arise—for example, genes which are not probed on the microarray or are ambiguous, the instance may still be scored using only one evidence type.When the final ranking score is derived from human and mouse data, the relative contribution of mouse tissue datasets relative to the human datasets can be adjusted by the user.

Modified *z*-scores for all RNA sequencing and microarray datasets were calculated as shown in Equation (2):

(2) $$z_{e\in E}=\frac{\mathrm{0.6745}\cdot (e-\overset{E}{¯})}{\mathrm{median}(|e-\overset{∼}{E}|)},$$

where *E* denotes a set of normalized expression values in an experiment, with individual elements *e*; $\bar{E}$ is thus the mean value of *E* and the denominator is the median absolute deviation, where *e* is an individual element of *E* and $\overset{∼}{E}$ is the median of all elements in *E.*

The modified *z*-scores enable the transformation of non-normally distributed gene expression data and measure how each data point differs from the typical observations within the dataset. This transformation serves both to aid the prioritization and to facilitate better comparability between microarray datasets, as it has been suggested that rank-based transformations of microarray data alleviate some of the issues associated with comparing cross-platform, cross-laboratory data (Irizarry *et al.*, 2005).

### 2.3 Benchmarking dataset

The performance of the application was evaluated using a set of 1000 gene-disease associations, which was generated using the ‘The Human Phenotype Ontology’ (HPO) (Kohler *et al.*, 2014) dataset as a source for disease genes and associated phenotypes. The HPO is a curated ontology, organizing human disease phenotypes described in the Online Inheritance In Man (OMIM) database (URL: http://omim.org/), as well as Orphanet (URL: http://www.orpha.net) and medical literature in a structured, controlled vocabulary. This enabled the generation of a large dataset while bypassing any inaccuracies that can arise from the lack of precision when text-mining unstructured entries in OMIM (which sometimes describe legacy or historical information).

A sub-group of all HPO phenotypes was selected based on the following criteria: high term specificity (defined as the distance of a term from the root of the ontology); terms which could be unambiguously mapped to tissues through axiomatic links to an anatomical ontology (Golbreich *et al.*, 2006, Kohler *et al.*, 2013, (Hoehndorf*et al.*, 2010) or manual assignment. A total of 2922 distinct disease genes were found to be annotated as associated with HPO diseases and were subsequently matched with selected phenotypes. Phenotypes, where a frequency modifier for a disease is denoted as ‘very rare’ or in the cases where the frequency modifier is given as a percentage, <2% of all cases reported, were not considered. Finally, from the resulting data, 1000 disease-genes associations were selected at random for testing (Supplementary Data 1).

In order to ascertain how tissue selection affects prioritization with GeneTiER, diseases with a distinct, localized phenotype were categorized using Disease Ontology, using definitions which are descendants of the term ‘disease of anatomical entity’ (DOID:7). 500 disease–gene associations were selected for testing (Supplementary Data 2).

### 2.4 Implementation

**Fig. 1.**
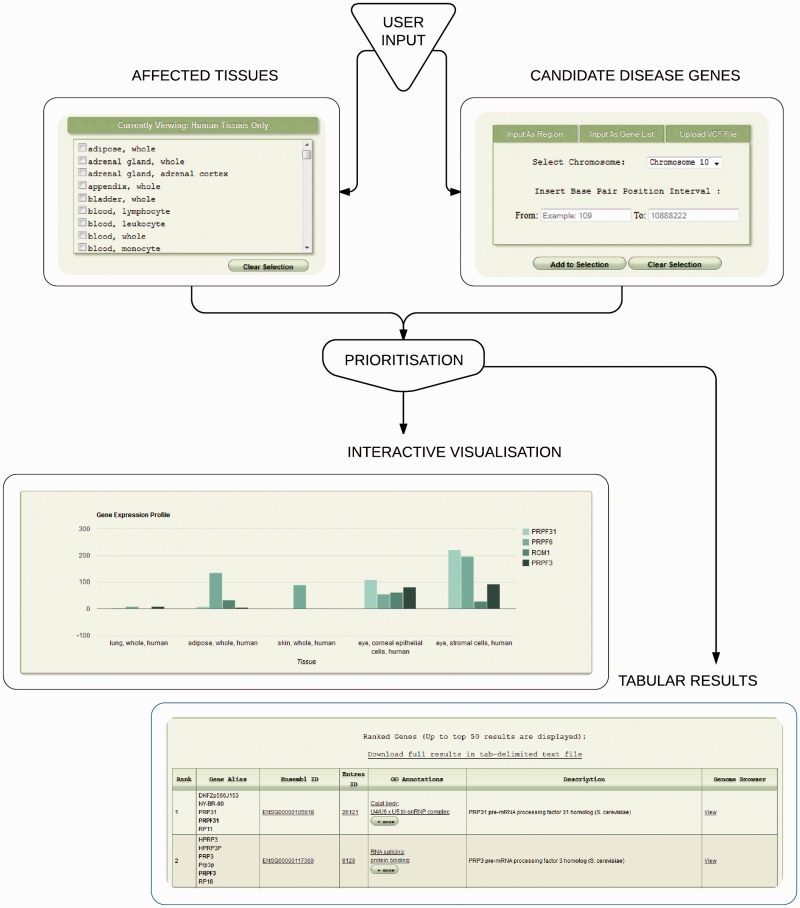
Overview of GeneTIER implementation. The web-based interface allows the user to supply candidate disease genes to prioritize and to select affected tissues. Top prioritization results are returned in a tabular form and are available to visualize and compare using an interactive chart. Full results are available for download

The implementation is accessible through a web-based interface (Fig. 1), which was constructed to HTML5 specification, utilizing JavaScript throughout; JavaScript browser support is thus required. Currently, the user may supply candidate disease genes in the following formats: a single or multiple genomic region(s), specified by chromosome and interval boundaries; a pre-filtered VCF file containing potentially deleterious variants (enforced by file size limit); a candidate gene list in a delimited format, accepting several commonly used delimiter types. The gene list can be composed of Ensembl (Flicek *et al.,* 2014), Entrez (Maglott *et al.*, 2011) or Refseq (Pruitt *et al.*, 2014) accession numbers, HGNC-approved gene names (Gray *et al.*, 2013); common aliases or any combination of thereof may be used as input. GeneTIER performs automatic conversions between human genes and their mouse orthologs, as well as optionally resolving ambiguous input gene aliases and identifiers based on multiple criteria. Up to 100 top results can be viewed directly in the browser and the entire analysis can be downloaded as a text file. GeneTIER is freely available at http://dna.leeds.ac.uk/GeneTIER/.

## 3 Results

The algorithm used by GeneTIER assumes that a disease gene’s expression tends to be significantly higher in affected tissues compared with unaffected tissue. To test the generality of this assumption, we retrieved expression values from our database for all genes in our training set (see Section 2) and performed a two-sample Kolmogorov–Smirnov test for non-normally distributed data, using an alternative hypothesis that the cumulative frequency distribution function of modified scores from unaffected tissues lies below that of modified scores from disease-associated tissues. For RNA-sequencing data this resulted in statistic *D* = 0.1517, with respective *P*-value < 2.2e^−16^ and for microarray data *D* = 0.1334, *P*-value < 2.2e^−16^.

The performance of GeneTIER was assessed on a dataset comprised of 1000 known associations between disease genes and tissues expected to be affected by each gene’s dysfunction. For each disease gene, four sets of random genes were generated, comprising 50, 100, 200 and 500 genes. The disease genes were prioritized against the genes in the randomly generated gene sets and the results analyzed using ROC analysis. ROC curves provide a way to visualize and compare classifier performance. Here, the candidate gene prioritization algorithm can be viewed as a non-binary scoring classifier, where disease-linked genes are positive instances and other candidates are negatives. The values—or ranks—from the classifier output can be converted into binary positive and negative scores using cut-off thresholds. Thus, a confusion matrix can be calculated for every integer rank cut-off value from which comparison metrics, such as sensitivity and specificity values are derived. ROC graphs allow the visualization of sensitivity versus 1-specificity. The line running from the origin (0,0) to the maximum point of 1,1 (*Y* = *X*), which corresponds to an area under the curve (AUC) of 0.5, represents a performance that is no better than random predictions. Points on a ROC curve that occur above this line represent an algorithm with better than random classifier performance, while those below the line have worse than random results, i.e. a bias toward classifying positives as negatives. An algorithm with an AUC of 1 represents perfect classifier performance.

**Fig. 2.**
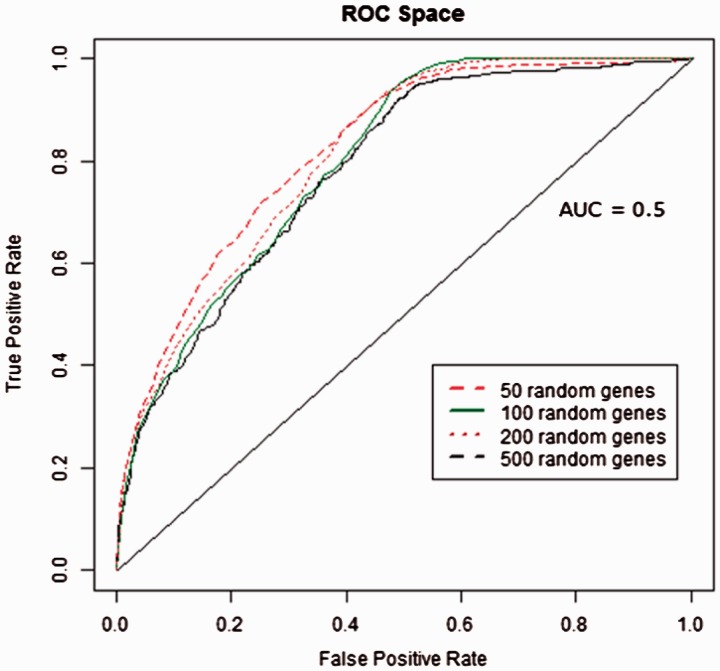
ROC curve showing classifier performance on different size input generated using disease genes from the benchmarking dataset (see Section 2)

Figure 2 shows the resultant ROC graphs while Table 1 shows the corresponding AUC scores. This analysis suggests that the algorithm’s performance was inversely related to the number of non-disease genes in the analysis, but does not decline in a linear manner. In fact, the differences in performance when assessed on candidate lists consisting of 100, 200 or 500 candidates are minor and do not suggest that the maximum candidate gene list size will be encountered in typical gene mapping experiments. Overall, the obtained AUC values are sufficiently high to suggest that disease genes are typically ranked higher than the randomly selected genes in each data set by this algorithm.

**Table 1.** AUC scores for classifier performance when assessed using 1000 known disease genes

Random gene sample size	Area under the ROC curve
50	0.83
100	0.80
200	0.81
500	0.78

**Fig. 3.**
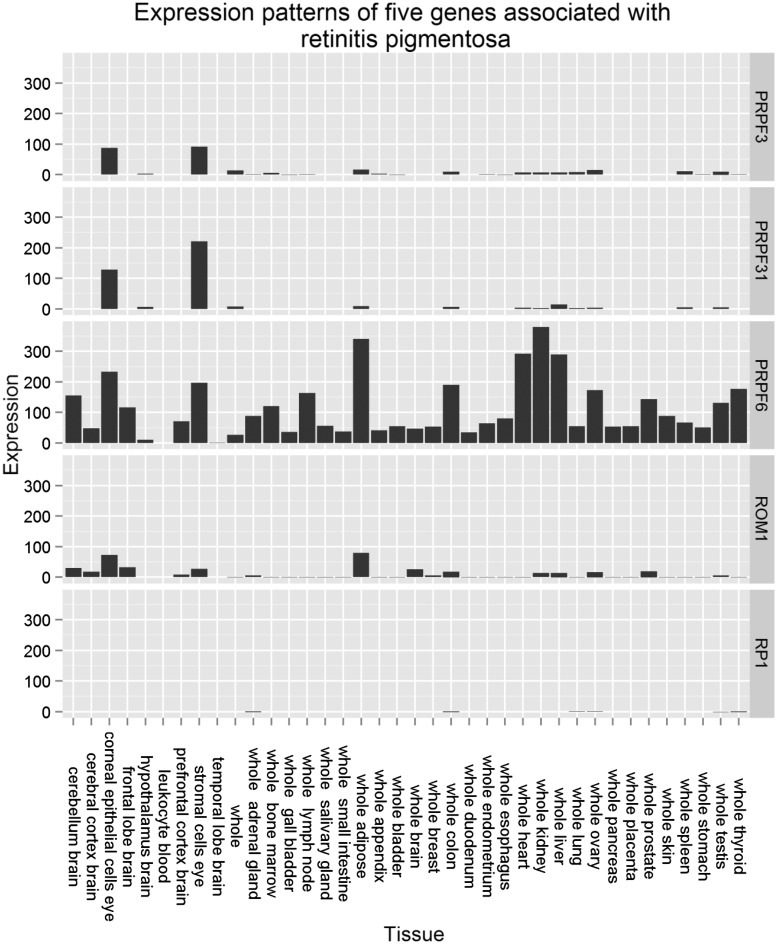
Expression profiles of PRPF3, PRPF31, PRPF6, ROM11 and RP1 genes, associated with retinitis pigmentosa (OMIM:610282) across a selection of tissues, RNA sequencing data

In order to illustrate the circumstances where our methodology either failed or succeeded, we used a case study of the global expression patterns for genes implicated in retinitis pigmentosa (OMIM:610282), a degenerative eye disease causing severe vision impairment. Figure 3 shows the expression profiles across multiple normal tissues of five disease genes known to underlie retinitis pigmentosa, while Tables 2 and 3 show the summary of the mean ranks obtained using our method. The genes PRPF3 and PRPF31 show distinct, tissue-specific expression in eye tissues with negligible expression in non-ocular tissue; disease genes with similar expression profiles are ranked very highly by our methodology. While PRPF6 is also highly expressed in eye tissues, unlike PRPF3 and PRPF31 its expression is not limited to ocular tissues, resulting in a reduced, but still strongly suggestive ranking. ROM1 is expressed in a number of non-ocular tissues as well as in corneal epithelial cells but still ranked highly. This was in spite of its lower expression in corneal epithelial cells than that of the PRPF genes and comparable expression in adipose tissue. Unsurprisingly, in view of its ubiquitously low expression levels, the methodology failed to identify PR1.

**Table 2.** Mean ranks and standard deviations of five case-study genes shown in Fig. 3

Gene	PR1		ROM1		PRPF6		PRPF31		PRPF3	
Input size	Mean rank	Standard deviation	Mean rank	Standard deviation	Mean rank	Standard deviation	Mean rank	Standard deviation	Mean rank	Standard deviation
50	34.7	11.03	8.16	11.7	8.8	4.2	2.9	1.78	5.4	3.01
100	66.03	8.9	17.07	6.38	22.7	5.53	4.13	2.21	7.1	2.54
200	172.07	5.75	53.87	6.47	28.33	3.20	20.3	3.91	28.0	4.08
500	288.11	13.33	67.4	10.03	140.65	15.07	39.24	4.45	41.65	6.51

Each gene was ranked 30 times against a set of 50, 100, 200 and 500 randomly generated genes

**Table 3.** Mean reciprocal ranks of five case-study genes assessed against a set with 50, 100, 200 and 500 randomly generated genes; 30 replicates

Mean reciprocal rank					
Input size	PR1	ROM1	PRPF6	PRPF31	PRPF3
50	0.78	0.09	0.09	0.08	0.08
100	0.66	0.17	0.23	0.04	0.07
200	0.86	0.27	0.14	0.10	0.14
500	0.58	0.13	0.28	0.08	0.08

In order to ascertain whether our method is more appropriate for certain disease types, we analyzed how well GeneTiER performs across a range of tissues. Supplementary Figure S1 highlights that GeneTiER can accurately prioritize genes across most tissue categories, recognizing endocrine and integumentary system-specific genes particularly well, with 73 and 76% of disease genes, respectively, ranked in the top 10. However, genes in the sensory category, comprising mostly eye-related disorders, ranked poorly.

The eye is a complex organ with many specialized tissue types. While the GeneTiER database contains expression data from corneal epithelial cells, stromal cells and murine lens, these do not encompass all the diverse cell types present in the eye. Consequently, diseases of the eye that do not affect these tissues will not be correctly prioritized by our approach. Conversely, diseases affecting vision can be neurodegenerative in nature, in which case the causative gene may not have a function in the eye.Finally, we have considered which data type—RNA-Seq or microarray—enabled more accurate prioritization results. We considered all diseases where both RNA-Seq and microarray data were available for all the identified affected tissue types. Supplementary Figure S2 shows the ROC curves obtained when the dataset was prioritized together with 100 random genes. The difference in performance between RNA-Seq and microarray data is minimal, with RNA-sequencing data giving better results (ROC 0.80 versus 0.78), but slightly worse than the combined score approach (ROC 0.81).This is in concordance with a recent study by Wang *et al.* (2014), who found that while more differentially expressed genes identified by RNA-sequencing than microarray studies could be verified by qPCR, the gain was mostly from the improved quantification of low abundance transcripts.

Furthermore, while sequencing data do provide a small improvement over microarray data in prioritization, this is offset by a more limited public availability of sequencing datasets. There are currently 41124 microarray datasets deposited in Array Express database in contrast to only 5745 RNA-sequencing experiments (accessed 01/03/2014).

## 4 Discussion

While a number of methods have been previously employed for the prioritization of candidate disease genes, none are universally applicable. These methods tend to rely heavily on prior knowledge about the disease, phenotype and/or genes, making them unsuitable systems for classifying novel and/or poorly characterized genes. The best performing methods have been shown to rely on a variety of information sources to compensate for inadequacies in knowledge in any single domain. However, there is value to be found in approaches which distance themselves entirely from the ‘guilt-by-association’ principle used by these methods, which have an inherent bias toward well characterized genes.

The wide range of open access gene expression data can serve to overcome the limitations of prior knowledge-based approaches by substituting gene- or disease-specific information with tissue-specific experimental data. However, implementations of this approach often exhibit certain limitations. Some implementations prioritize candidate disease genes based on co-expression with genes already known to be associated with the disease in question. This requires the user to supply ‘seed’ genes, thus making the assumption that pre-existing knowledge about the disease is either available or relevant to the particular disease phenotype. This may not be the case; for example, OMIM currently contains several thousand disease entries for which no contributing genes are yet known.

Conversely, a number of network-based methods prioritize genes under the assumption that a disease gene will exist in a local network of genes which are highly differentially expressed between affected and unaffected tissues. However, while this type of approach can be successful in disease-specific studies, it is difficult to generalize for wider use, due to its reliance on differential expression datasets between disease and control tissue samples. Some implementations overcome these difficulties by requesting the user to supply their own data. However, this can be seen as a contradiction to the major aim of computational candidate prioritization which is to reduce the experimental burden on a researcher, rather than requiring the user to perform further studies. In this study, we present a novel application for candidate disease gene prioritization that aims to address the shortcomings discussed above. GeneTiER does not require the user to have any prior knowledge of the disease, other than the ability to unambiguously identify affected tissues. The application has been specifically designed to require only minimal user input, and takes care of conversions between a variety of commonly used gene identifiers and between human/mouse orthologs.

We have taken advantage of both microarray expression and RNA sequencing data available in the public domain to create an extensive tissue-specific expression database that can support a wide variety of gene prioritization queries. Organs are made up of many functionally diverse tissue cell types and this can be reflected in the experimental data. Therefore, we have striven to collate data from multiple, distinctive datasets, to enable the user to make tissue cell type-specific queries which are not supported by many of the popular databases.

Many popular gene prioritization methods that rely on prior knowledge about a disease use either text-mining approaches or Gene Ontology annotations to score candidates based on relevance to query. Currently, there are still over a thousand human genes with no available GO annotations and many more with ‘shallow’ annotations. While this presents a problem for disease gene inference by similarity, the method described here would not be any less applicable. For example, at the time of writing, no Gene Ontology annotations have yet been ascribed to the human *CDR1* gene, known to contribute to paraneoplastic cerebellar degeneration (OMIM:302650). This gene shows localized expression in brain tissues, in particular in the cerebellum, and as such is scored highly by our method, whereas approaches reliant on prior knowledge are likely to fail. However, direct performance comparisons between gene prioritization tools are difficult—without cross-validation, any prioritization on known disease genes is meaningless, and to the best of our knowledge, no web gene prioritization application provides a performance assessment mode.

While we have shown that GeneTiER is capable of accurate disease gene prioritization through ROC analysis (with AUC values of up to 0.83), it should be noted that the disease gene is rarely ranked first in the output. This ranking should therefore be used as a guide to the order in which candidate genes should be analyzed further. Even so, it must be noted that not all disease gene expression patterns conform to the assumptions underlying our model. For example, some disease genes show universally high or low gene expression across all tissues (see RP1 in Fig. 3). Indeed, aberrant activation of genes which are normally repressed can result in a disease phenotype, as is the case in many cancers. However, in order to detect these patterns, the differential expression change must be observed between the normal and affected states. While including differential expression data from normal and affected patients would no doubt improve GeneTiER performance, public availability of such data is mostly limited to a small number of well-studied diseases and therefore would enhance the results for only a small proportion of cases.

Furthermore, as Oellrich and Smedley (2014) note in their analysis, the site of gene expression and the visible phenotype do not always coincide. Consequently, the limitations of this method must be understood and taken into consideration when examining the final gene rankings. This is especially true where the link between tissue and phenotype may not be immediately obvious. For example, congenital dysfibrinogenemia (OMIM:616004) is a blood clotting disorder caused by defective fibrinogen genes *FGB*, *FGG* and *FGA.* Circulating factors affecting blood clotting are synthesized by hepatocytes, and indeed, our data show that fibrinogen genes are highly and exclusively expressed in the liver (Supplementary Fig. S3a). However, GeneTiER would not identity these disease genes if the user failed to take this into account and selected blood, rather than liver, as the affected tissue.

Narcolepsy-cataplexy (ORPHANET:2073) is a sleep disorder with multiple causative genes identified. GeneTiER scores a number of these highly, for example MOG and ZNF365, due to localized expression in parts of the brain (Supplementary Fig. S3b). However, the disease can have an autoimmune component and in some patients the phenotype has been attributed to the loss of neurons in the hypothalamus due to autoimmune attacks. Consequently, our methodology fails to identify histocompatibility genes *HLA-DQB1* and *HLA-DRB1* as causative genes for the disease and may find other phenotypes arising from heterogeneous causes challenging (Supplementary Fig. 3c).

Similarly, GeneTiER will not be able to identify disease genes that are expressed exclusively in tissues not present in our dataset. Similarly, diseases caused by genes that are expressed in response to either an environmental stimulus or within a short development time frame will not perform well if the appropriately stimulated tissue is absent from our dataset.

In spite of these challenges, there are numerous cases where the observed phenotype correlates with the site of expression exceedingly well. For example, renal tubular dysgenesis (OMIM:267430) is characterized by a congenital abnormality of the kidneys, with low amniotic fluid during pregnancy. The protein associated with the disease, *REN*, is produced mostly by juxtaglomerular cells of the kidney. The data in GeneTiER database agree with this, showing elevated expression in the kidney, as well as a secondary major site of expression in the placenta (Supplementary Fig. S3d).

Our testing indicates that our gene prioritization method is capable of meaningful candidate gene prioritization and performs strongly in a substantial proportion of cases tested. GeneTiER aims to highlight genes with tissue-specific expression patterns to the user from among other candidate genes in their dataset, and as such will perform best for diseases with distinct, localized phenotypes. Nevertheless, a broad selection of tissues allows for scoring of complex phenotypes affecting any combination of tissues. We believe GeneTiER offers great utility value to the research community and can effectively supplement the *in silico* toolbox of any researcher.

## Funding

This work was supported by grants from Sir Jules Thorn Charitable Trust (Grant 09/JTA); and the MRC (MR/L01629X/1).

*Conflict of Interest*: none declared.

## Supplementary Material

Supplementary Data
