# Supplementary material for: GeneTIER: prioritization of candidate disease genes using tissue-specific gene expression profiles
Source: Bioinformatics. 2015 Apr 9;31(16):2728–35. doi: 10.1093/bioinformatics/btv196 (PMC4528628; doi:10.1093/bioinformatics/btv196)
Supplement: Supplementary Data [file supp_31_16_2728__index.html]

GeneTIER: prioritization of candidate disease genes using tissue-specific gene expression profiles — GeneTIER: prioritization of candidate disease genes using tissue-specific gene expression profiles — GeneTIER: prioritization of candidate disease genes using tissue-specific gene expression profiles — Supplementary Data 

# GeneTIER: prioritization of candidate disease genes using tissue-specific gene expression profiles

## Supplementary Data

files

**Files in this Data Supplement:**

- Supplementary Data - zip file
