# Supplementary figures and images for: GeneTIER: prioritization of candidate disease genes using tissue-specific gene expression profiles
Source: Bioinformatics. 2015 Apr 9;31(16):2728–35. doi: 10.1093/bioinformatics/btv196 (PMC4528628; doi:10.1093/bioinformatics/btv196)

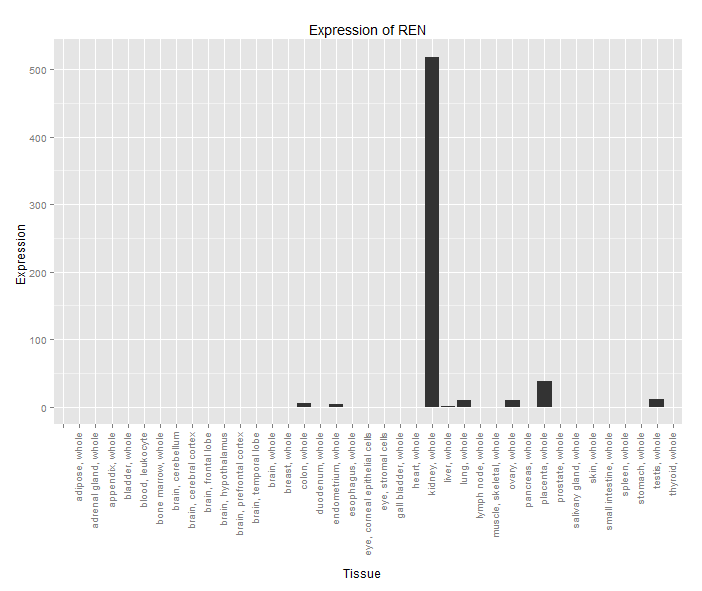

Supplement: Supplementary Data [file supp_btv196_suppl_data.zip › supplementaryfigure3d.png]

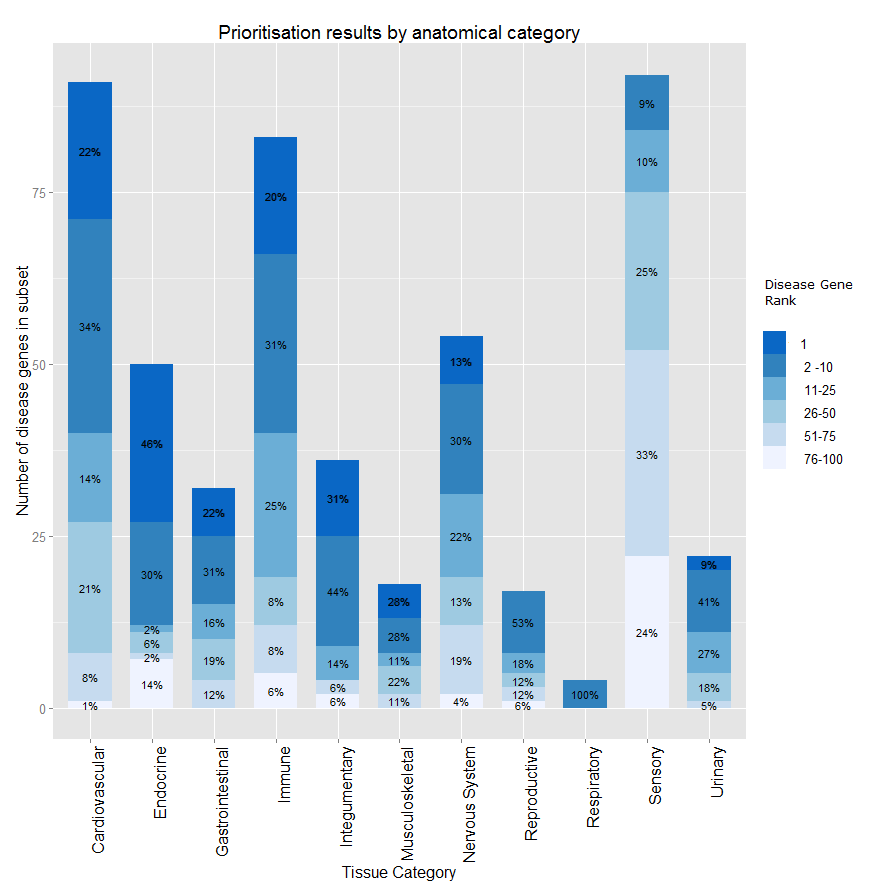

Supplement: Supplementary Data [file supp_btv196_suppl_data.zip › supplementaryfigure1.png]

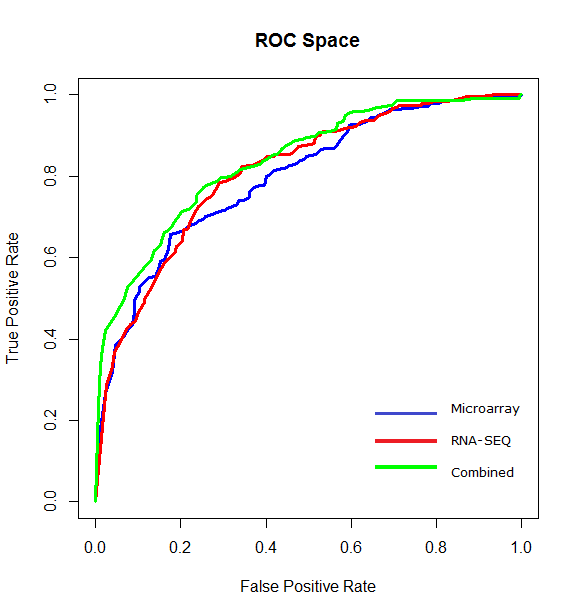

Supplement: Supplementary Data [file supp_btv196_suppl_data.zip › supplementaryfigure2.png]

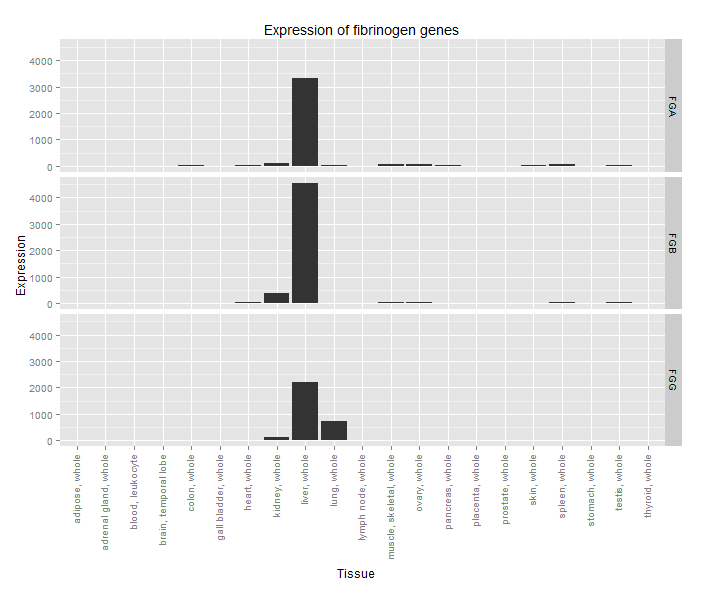

Supplement: Supplementary Data [file supp_btv196_suppl_data.zip › supplementaryfigure3a.png]

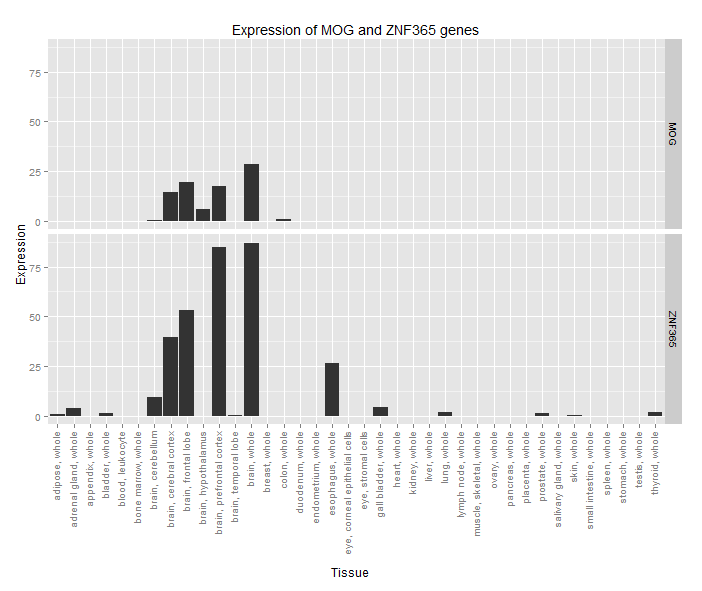

Supplement: Supplementary Data [file supp_btv196_suppl_data.zip › supplementaryfigure3b.png]

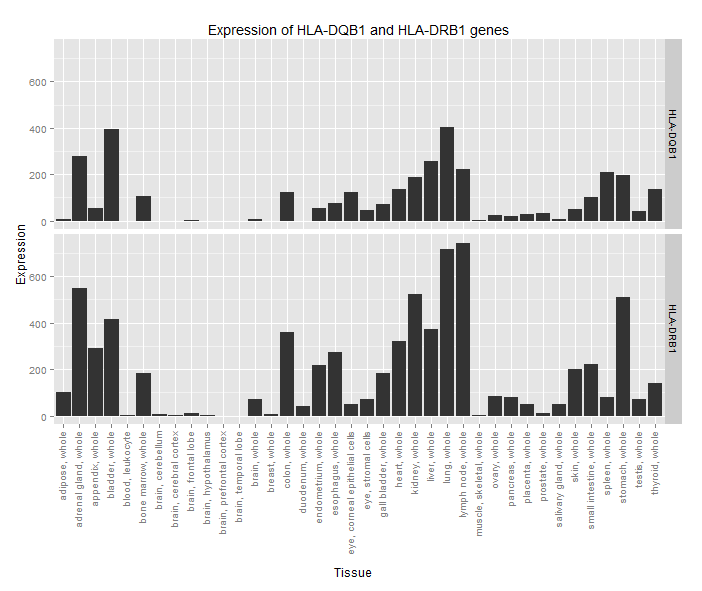

Supplement: Supplementary Data [file supp_btv196_suppl_data.zip › supplementaryfigure3c.png]
